# Determining factors affecting Filipino consumers’ behavioral intention to use cloud storage services: An extended technology acceptance model integrating valence framework

**DOI:** 10.1016/j.heliyon.2024.e26447

**Published:** 2024-02-16

**Authors:** Gerlyn C. Altes, Ardvin Kester S. Ong, Josephine D. German

**Affiliations:** a School of Industrial Engineering and Engineering Management, Mapúa University, Manila, Philippines. 658 Muralla St., Intramuros, Manila, 1002, Philippines; b School of Graduate Studies, Mapúa University, Manila, Philippines. 658 Muralla St., Intramuros, Manila, 1002, Philippines; c E.T. Yuchengo School of Business, Mapúa University. 1191 Pablo Ocampo Sr. Ext., Makati, Metro Manila, 1205, Philippines

**Keywords:** Cloud storage, Structural equation modeling, Technology acceptance model, Valence framework

## Abstract

Cloud Storage (CS) is a service that digitally stores, remotely manages, backs up, and renders internet-accessible data. However, despite its known benefits compared to traditional storage devices, this service is not widely used in developing nations such as the Philippines. This study integrated the Valence Theoretical Framework into the Extended Technology Acceptance Model (ETAM) to evaluate the influence of twelve variables on Filipino consumers' behavioral intention (BI) toward adopting CS services. The data is gathered through an online survey. Structural Equation Modeling was employed to examine the responses of 431 cloud users, mainly students and working professionals. Results showed that Perceived Benefit and Perceived Usefulness were the strongest determinants of BI. The Job Relevance was also found to be a significant factor. Therefore, CS providers should find additional ways to make their offerings more beneficial for the daily tasks of students and working individuals. Furthermore, considering the substantial influence of Perceived Risk and Subjective Norms on BI, CS providers must strengthen their security measures to boost users' trust in their services. Consumers who receive excellent service are likely to give positive reviews, which can be helpful to individuals who might also be considering purchasing CS for their data. Although the focus of this study is CS services, this can also serve as a reference when analyzing the BI of consumers concerning the adoption of other novel technologies applied in various sectors, including education, e-commerce, healthcare, and more.

## 1 Introduction

Data is a collection of information and can come from different sources. It can originate from documents, camera recordings, books, logs, or practically anywhere. It is important because it can be used to analyze trends, build insights, make projections, make decisions, and develop new products and services [1,2]. Data is typically stored on thumb drives, optical storage devices such as CDs, DVDs, and Blu-ray Discs, and internal or external drives such as hard disk drives and solid-state devices. These physical storage devices allow users to access their data without an internet connection and securely transmit information to one another by passing along their media. The issue with physical storage media is that these have limited storage capacity, they can be misplaced or lost, and they can be broken if dropped [3,4]. Consequently, as time passes, the amount of data generated and collected increases continuously. This scenario necessitates the need for storage space to save these data. Due to the massive amount of data, setting up server computers to store it is essential. However, setting up server computers is highly expensive. An alternative to this is the use of cloud storage [2].

Cloud Storage (CS) is a service that keeps data on remote servers that are hosted, managed, and protected by a third-party service provider. Users send data to servers through the internet, which will be stored on a virtual machine on a real server. The information stored on the remote servers is always accessible via public or private internet connections. Users can access their data anytime from web portals, browsers, and mobile apps via an application programming interface [5].

CS has greater capacity and reduces the cost of purchasing and maintaining a physical server. It is more advantageous than traditional offline storage since CS providers have readily available backups. Files can be mobile and accessible on different electronic devices and locations [6]. By eliminating the requirement for data storage infrastructure on the business premises, CS enables companies with massive data storage requirements to save significant space and money. In addition, businesses can instantly increase or decrease the CS they can access as their storage needs vary. Furthermore, the cloud allows employees to collaborate and work with coworkers remotely and outside of regular business hours. Seamless document collaboration is enabled by granting users easy access to the most recent version of the files [7].

Despite the benefits of CS services, it is not widely used, particularly in developing nations such as the Philippines. According to the study conducted by the Statista Research Department in 2021, only 11% of 870 adult respondents in the Philippines have experience in using CS [8]. In contrast to neighboring developing nations such as Malaysia, CS technology is widely adopted and even invested in. In the same year, a survey conducted by Nutanix Malaysia, a cloud computing company, revealed that 58% of Malaysians invested in private cloud as demand for digital solutions in the workplace has increased due to the pandemic [9]. Survey results revealed that CS adoption among Filipinos is relatively low. The survey results prompted the question of how well Filipinos accept and adopt this technology and what factors affect Filipino consumers’ behavioral intention to use CS services.

There are a few available studies regarding cloud computing and CS adoption in the Philippines; however, most are limited to particular groups such as students and micro, small, and medium enterprises (MSMEs) and need more representation of other groups of the population of the Philippines. A study conducted by Tubay [6] on university students in the Philippines using the Unified Theory of Acceptance and Use of Technology (UTAUT) and Partial Lease Square – Structural Equation Modeling (PLS-SEM) revealed that social influence and performance expectations affected the intention to utilize CS systems. Students are likely to use a CS system if they perceive that their individual or group-based coursework can be managed and completed effectively and efficiently through CS. In addition, a significant and positive social influence suggests that students are likely to use CS if their peers and teachers use it and expect them to use it as well. The researcher then suggested that the concerned university maintain its subscription to a CS system and promote maximizing its use because of its popularity among a large academic community. Furthermore, the students expressed their intention to utilize the CS system due to their perception of enhanced academic productivity and the concurrent growth of the university population.

In Malaysia, however, factors for the adoption of cloud computing among higher education students differ from the university students in the Philippines. Amron and Noh [10] conducted a study utilizing the Technology Acceptance Model (TAM) and Partial Least Squares Structural Equation Modeling (PLS-SEM). Their findings indicate a substantial and positive correlation between perceived usefulness and perceived ease of use and the behavioral intention to utilize cloud computing among students, teachers, and staff in higher education institutions in Malaysia. The security and learning environments were also observed to have a positive and substantial effect on the behavioral intention to utilize cloud computing. Due to the pandemic, the learning environment has contributed to adopting cloud computing, as students and instructors must implement distance learning and use online resources.

In a study on MSMEs in the Philippines using the Technology Organization Environment (TOE) framework and Multiple Linear Regression (MLR), Matias and Hernandez [11] found that competitive pressure and regulatory support affected firms to adopt the cloud. The adoption of cloud computing in businesses is independent of variables such as perceived benefits, IT capability, and business issues. Their research revealed that the management acknowledges technological progress as advantageous and beneficial to the organizations. Management must procure or cultivate a proficient group of IT personnel to successfully deploy new technologies.

Song et al. [12] examined the adoption of Public CS Services (PCSS) by non-organizational individuals in general in a study conducted in South Korea. It was hypothesized that the degree to which individuals employ cloud technology could differ depending on their usage. Through the application of UTAUT and PLS-SEM, they successfully ascertained a substantial distinction in the social influence exerted on nonusers and users of the cloud. In contrast to nonusers, cloud users did not exhibit a positive intention to utilize PCSS as predicted by social influence. That said, no other substantial differences existed between the two categories. Nevertheless, they displayed significant similarities. The habit was the most precise predictor of the inclination to utilize cloud services for both cloud users and nonusers; hedonic motivation did not emerge as a significant factor for either cohort. Performance expectations influenced the implementation of PCSS in both categories. Positive intention to utilize PCSS was determined by expectations of effort from PCSS users but not by expectations of action from nonusers.

A good understanding of the needs and acceptance of individuals would be helpful to the betterment of technology, its users, and developers [13]. As evident in the background discussion, a lot of developers and users are considering Cloud Storage services – especially in the Philippine business and education sector [[6], [7], [8], [9]]. However, only few studies have been evident to provide insights into the behavioral intention and actual use of this system. Hence, to understand factors affecting Filipino consumers' behavioral intention to use CS services, this study used TAM which is a well-established model for assessing technology adoption. To further explore possible factors affecting behavioral intentions, Subjective Norms, Experience, Job Relevance, and Voluntariness were added to the usual components of the TAM. Furthermore, to address TAM's lack of consideration of the positive and negative attributes of technology, the Valance Framework was integrated with it. Thus, the following constructs were added to the extended TAM: Perceived Ubiquity, Perceived Benefits, Perceived Risk, and Perceived Cost.

According to recent publications spanning the past decade, TAM is widely used in e-commerce, online banking, and social networks. However, it is less commonly used in domains such as Cloud Computing and Augmented Reality. Developed countries tend to have a prevalence of research studies utilizing TAM in comparison to emerging ones [14]. Furthermore, most of the literature that was reviewed for this study rarely incorporated the Valence Framework to the TAM or extended TAM (ETAM), and further discussion is discussed in the succeeding section.

Therefore, this study contributes to the limited literature that integrated Valence frameworks into the ETAM and justified through structural equation modeling (SEM) to analyze consumer adoption of CS services in a developing nation, particularly the Philippines. Finally, while this study primarily focuses on Cloud Storage services, it may also be utilized as a reference for analyzing consumer behaviors in other domains, including e-Learning, online banking, e-commerce, mobile commerce, social networks, healthcare, and others.

## 2 Literature review and hypothesis development

### 2.1 Cloud storage and cloud computing

CS is a service architecture in which data is transmitted and kept remotely on remote storage systems. On these remote storage systems, data is backed up, maintained, and made available to users over the network. CS users pay monthly, depending on their consumption rates, to CS providers. CS has four deployment models: Private, Public, Hybrid, and Community cloud. A private cloud infrastructure is established and preserved exclusively for a designated organization or enterprise. A public cloud is a service commercially accessible to the general public via the Internet. A hybrid cloud combines private and public clouds with scalability, cost efficiency, security, and flexibility. A community cloud shares similar storage among organizations and companies with the exact requirements and interests [15,16].

CS is part of cloud computing. Cloud computing exists to give users a more effective and efficient data access experience. Wherever the users are, they can readily acquire data and use it to their advantage. Cloud computing is growing more sophisticated as internet technology advances and consumer access becomes more convenient. As a result, users are capable of synchronizing data with any device. Users no longer have to keep data on a local computer; instead, data will be saved to the cloud and safeguarded by the CS service provider [17].

Cloud computing utilizes various technologies, including distributed storage and virtualization, to process data for various tasks. Cloud computing comprises three different service levels that operate primarily on a "Pay-per-use" model: Software as a Service (SaaS), Platform as a Service (PaaS), and Infrastructure as a Service (IaaS). The SaaS is a cloud computing service enabling users to access vendor-provided software applications through the cloud. SaaS providers include Google Chrome or Internet Explorer. PaaS is a cloud computing service that enables consumers to develop, manage, and execute applications on the cloud. Examples of PaaS include Microsoft Azure and Google Cloud. Unlike SaaS and PaaS, IaaS is a cloud computing service where a vendor offers users access to its hardware resources, such as storage, servers, and networking devices. Amazon Web Services (AWS), Elastic Compute Cloud, and Simple Storage Services (S3) are examples of IaaS. CS cannot be limited explicitly as part of any of the service layers because it is incorporated in all of the layers. There are many CS providers, so to entice businesses and individuals, cloud providers offer a portion of the capacity free of expense. For example, Dropbox offers a complimentary storage capacity of up to 6 GB through social network connection and invites acquaintances to join the platform. Similarly, Google Drive, Box, Amazon, and Apple Cloud offer a maximum of 5 GB of complimentary storage space. CS is beneficial for businesses and various uses, even for personal use [6,18,19].

### 2.2 Research models

A Bibliometric Study by Al-Emran and Granic [67] revealed that during the past thirty years, a considerable quantity and variety of theoretical perspectives have been introduced in an attempt to provide insight into the factors that influence technology and its usage. The TAM has emerged to be a powerful and concise framework for understanding the factors that influence the adoption of a technology; and it focuses on two key beliefs: Perceived Ease of Use and Perceived Usefulness. As per the aforementioned study [20], TAM demonstrated considerable potential for implementation across diverse domains, including (but not limited to) the extensively researched areas of e-commerce and online banking, e-Learning for education, cloud computing, and augmented reality. Furthermore, TAM has undergone extensive integration with numerous established theories and models across disciplines, which has enabled the development of integrated models to help in technology assessment. TAM has frequently been combined with one model and two theories: the DeLone and McLean IS Success Model, the UTAUT theory, and the DOI theory. The derived integrated models have been recognized as reliable frameworks for organizing and conducting empirical research regarding the adoption of technology.

In contrast, the valence framework is a well-established theory that originates from economic and psychological theories in behavioral research. According to the theory, Perceived risk and benefit are the fundamental factors that influence consumers' decision-making. When deciding to purchase a product or service, consumers aim to maximize the positive valence (perceived benefits) and minimize the negative valence (perceived risks). Moreover, the theory provides a rationale for how the positive and negative attributes can predictably influence the decision to adopt or use a certain product or service [[21], [22], [23], [24]].

TAM places greater emphasis on user and technology behavior than on the actions and mechanisms that may lead to goal attainment [25]. Although UTAUT addresses certain limitations of TAM by incorporating a diverse set of variables, including the impact of social contexts such as facilitating conditions and social influence, it is challenging to meaningfully categorize these variables due to their multifaceted implications. The valence framework addresses the disorganization issue caused by various unrelated variables by categorizing technological qualities into positive and negative valences based on their impact on the decision-making process [26,27]. Thus, incorporating the valence framework into the TAM would make this study more concise in terms of determining consumers’ behavioral intentions.

Reviewed related literature using TAM and the Valence Framework explaining consumer behaviors in different fields are summarized in Table 1. Based on the table summary, TAM and the Valence Framework are rarely used together. Therefore, this study would be beneficial for future studies that aim to evaluate technology adoption based on the established factors of the TAM, but would also want to consider the impact of positive and negative qualities of the technology on consumer behavioral intentions.

**Table 1:** Technology adoption in various fields using TAM/ETAM and Valence Framework.

Domain	Year	Research Model	Analysis Model	Findings	Reference
Digital Banking (DB)	2023	ETAM	PLS-SEM	Perceived Usefulness (PU) has significant impact on the Behavioral Intenton (BI)	[27]
eHealth systems	2020	ETAM	SEM	PU has significant impact on the BI	[28]
Education and Information Technology	2021	ETAM	PLS-SEM	Computer competencies (CC) and Computer self-efficacies (CE) with significant impact on the BI	[29]
Telemedecine	2020	ETAM	PLS-SEM	PU, Perceived Ease of Use (PEOU), Facilitating condition (FC), Trust positively affects BI. While the Technological Anxiety (TA), Resistance to Use (RC), Privacy (Pri) negatively affects BI.	[30]
Social Media Addiction	2023	ETAM	PLS-SEM	Usage Habit (UH) a strong predictor of addiction, as well as PEOU, PU.	[31]
Software and IT product for Home Energy Managament System	2020	ETAM	PLS-SEM	Environmental Responsibility (ER), Social Contribution (SC) affect PU, Innovativeness (IN) affects PEOU. Both PU and PEOU are significant to BI.	[32]
Digital competencies of librarions	2023	TAM	CFA, SEM	Librarians' digital abilities are critical and affect how they utilize and apply emerging technologies in handling and developing digital library services.	[33]
Cloud Computing (CC)	2020	TAM3	Independent sample *t*-test, Mmulti-Group Confirmatory Factor Analysis (MGCFA)	Although no findings which clearly show that CC acceptance and use is related with socio-cultural differences, Computer Anxiety (CANX) is more prevalent in Turkish students than UK students.	[34]
Cloud Computing Dependability	2022	ETAM	PLS-SEM	PU has significant impact on the BI. The usefulnes of CC has more effect on the intention to use software-*as*-a-service (SaaS)than to use platform-*as*-a-service (PaaS).	[35]
Drivers of Personal Cloud Computing	2022	ETAM	SEM	Perceived Cost (PC), Perceived Speed Access (PSA), and Technology Competency (TC) influence BI. A new technology that can render a great service a will be useless if not affordable.	[36]
Mobile payment system (MPS)	2022	Extended Valence Framework	Partial Least Squares (PLSc)	PB and Trust influences BI to use MPS. While, Perceived Risk (PR) has no impact to BI.	[21]
Blockchain (BC) Technology	2021	Extended Valence Framework; Technology Organisation Environment (TOE)	SEM	Technological Factors (TFc), Organization Factors (OFc), Performance Expectancy (PEx), and Trust affects BI of blockchain adoption.	[22]
Cross-border e-commerce (CBEC)	2019	Valence Framework	PLSc, Common method variance (CMV)	Uncertainty is significant for repurchase intention, especially among North American consumers. Buyers lack information on the products featured and on their performance qualities.	[23]
Adoption of eCommerce in MSME	2023	Valence Framework, DeLone & McLean (DM)	PLS-SEM	PB significantly affects MSME's BI	[24]
Cloud Computing (CC) Adoption	2019	TAM; Valence Framework	SEM	PU, PR, PC, PB influence BI	[36]

### 2.3 Technology acceptance model

The TAM by Davis and Olson [37] is a theory that models how users embrace and use technology. According to the theory, when consumers are presented with a new technology, various factors influence their decision regarding when and how to use it. In general, TAM emphasizes two principal factors, PU and PEOU, when analyzing the adoption of technology by individuals. PU is the extent to which an individual believes technology would improve their job performance. PEOU refers to the extent to which an individual believes utilizing a particular technology would be effortless. The literature demonstrates that TAM can reveal the variables influencing the decision to use new technology. This study employs TAM because it is appropriate for predicting technology acceptance factors. Furthermore, this theory permits testing external variables alongside the theory's two factors [10,38].

#### 2.3.1 Behavioral intention, perceived usefulness, and Perceive ease of use

TAM has been frequently referenced in contemporary scholarly studies as a highly regarded and extensively proven theoretical framework for explaining and predicting consumer behavioral intention (BI) toward various technological solutions. The TAM posits that the formation of BI is influenced by attitude, which can be assessed through the factors of PU and PEOU. BI refers to an individual's psychological reaction toward utilizing a particular system [39]. In essence, BI is a person's effort to engage in a particular behavior. The greater a person's intention, the more likely they will engage in the specific activity. Therefore, it was hypothesized that.

Hypothesis **(BI → AU)**: BI positively influences the actual usage of CS services.

TAM assumes that users' perceptions of its usefulness influence the intention to use new technology. PU relates to how users believe utilizing a particular technology will enhance productivity or performance. In other words, an individual's subjective evaluation of the PU of new technology will determine their acceptance of it. In contrast, the PEOU pertains to the extent to which users believe the system is simple and less complicated [40]. Therefore, it was hypothesized that.

Hypothesis **(PEOU → PU)**: PEOU positively influences PU.

Hypothesis **(PEOU→ BI)**: PEOU positively affects BI to use CS services.

Hypothesis **(PU→ BI)**: PU positively affects BI to use CS services.

### 2.4 Extended TAM

Venkatesh and Davis [41] extended the TAM by including new factors to improve its adaptiveness, explanatory power, and specificity. The added factors are social influence (image, subjective norms, and voluntariness) and cognitive (results demonstrability, job relevance, and output quality). The TAM2 is outperformed in both voluntary and forced situations. The only exception is subjective norms, which influence required settings but do not occur in voluntary settings [13]. Thus, it is suggested that TAM is utilized if the system or technology being evaluated is already established and has been utilized.

#### 2.4.1 ETAM and subjective norm

The original TAM did not include the Subjective Norm (SN). However, social psychologists know that a person's social context can alter their perception of unchanging physical objects. Venkatesh and Davis [41] understood its potential significance and hypothesized that SN influenced PU and intention to use in the TAM2. SN refers to the view of individuals who hold significant influence and play a determining role in shaping the behavior of others [42]. In other terms, SN refers to the influence of a person's social networks, including family and peers, on his or her behavior. When a user receives overwhelming influence from those around him or her to use technology, the user will be influenced to do so [43]. Thus, the statements mentioned above give rise to the following hypothesis.

Hypothesis **(SN → PU)**: SN influences PU.

#### 2.4.2 Experience and voluntariness

TAM2 also proposed that experience (EX) and voluntariness (VO) interact with SNs to shape PU and BI. EX refers to the interactive response to the stimulus of an object. The experience of using a product or service to which new technology has been employed can heighten awareness, familiarity, and comfort with the technology. Suppose a product with new technology becomes more prevalent in consumers' daily lives, and there is consistent growth in its user base, then familiarity with the technology will also rise. Thus, anyone who regularly uses a product incorporating new technology is predicted to increase its acceptance. On the other hand, VO refers to the voluntary utilization of a particular technology. The TAM2 model included VO, allowing voluntary utilization to control the interaction between social influences on technology adoption and behavioral intentions. VO can be categorized into two, namely environmental and consumer characteristics. The environmental characteristic posits that technology acceptance can be attributed to external environmental effects. VO remains unaffected by any potential biases or perspectives. The consumer's decision to utilize a product or service is influenced by their cognitive-driven voluntary motivation, which stems from an individual's internal intent, commonly referred to as intrinsic motivation [44]. Thus, the following were hypothesized.

Hypothesis **(EX → BI)**: EX positively affects BI to use CS.

Hypothesis **(VO → BI)**: VO positively affects BI to use CS.

#### 2.4.3 Job relevance

The concept of Job Relevance (JR) refers to an individual user's perception of the degree to which a specific system or technology aligns with their particular job duties [45]. The value of the set of activities that a system or technology can support within an individual's job determines the relevance of the system to the job. Thus, JR may be viewed as a cognitive decision that directly influences perceived usefulness and is not influenced by social interactions [46]. Hence, it can be hypothesized that job relevance affects perceived usefulness.

Hypothesis **(JR → PU)**: JR influences PU.

### 2.5 Valence framework of Behavioral Beliefs

The Valence Framework, developed by Peter and Tarpey [47], draws upon existing literature in economics and psychology to explain consumer behaviors. It posits that behavior is influenced by individuals' subjective evaluation of the potential risks (considered as negative utility) and rewards (considered as positive utility) associated with acquiring a particular item or service. These two essential features have a significant role in shaping consumer behaviors. Since consumers generally use a rational model in which they want to maximize their outcomes when making decisions, and since the valence framework is regarded as a superior model to understand the variety in consumer decisions due to its careful consideration of both rewards and risks, as well as uncertainty expectations, this study incorporated the valence framework into the TAM theory [48].

#### 2.5.1 Perceived ubiquity and perceived benefits

The positive utility component of the valence framework evaluates the extent to which adopting services can provide consumers with relative advantages. The capability of cloud computing services to be accessed and made available at any time and from any location signifies the ubiquitous nature of cloud computing. Cloud computing's ubiquitous nature empowers users to conveniently retrieve information or utilize applications using their devices, irrespective of their geographical locations. In contrast, the PB of cloud computing services is associated with the operational benefits an organization anticipates from adopting cloud computing. The benefits include enhanced mobility, efficient cost reduction, simple installation and maintenance, and internet-based data analysis. In addition, the PB of cloud computing includes cost savings, scalability, portability, and less software and hardware obsolescence. The PBs influence businesses' adoption of cloud computing. Therefore, the positive utilities PQ and PB were regarded as factors that could influence BI to adopt cloud computing [36]. Thus, the following were hypothesized.

Hypothesis **(PQ → BI)**: PQ positively affects BI to use CS services.

Hypothesis **(PB → BI)**: PB's positively influence BI to use CS services.

#### 2.5.2 Perceived cost

According to Changchit and Chuchuen [49], the primary advantage of adopting a cloud computing model is the potential for cost savings. No software installation, maintenance, or updates are required, which reduces IT costs. Cloud computing eliminates the need for expensive processors with large data storage capacities, expensive hard drives, Random Access Memories (RAMs), and operating systems. Lastly, a physical space to store equipment or for infrastructure facilities is unnecessary.

However, cloud computing is not always cheaper than traditional on-premises systems. Traditional IT resources, such as hard disk drives (HDDs), have experienced significant price drops over the past decade, with solid-state devices (SSDs) following closely behind. The vast majority of experts anticipate SSDs to be less expensive than HDDs shortly. Yet, CS prices have not decreased to the same extent, and some providers' storage prices may even increase [50].

In a study, Fisher [51] compared and calculated the cost-effectiveness of cloud services versus a more conventional self-reliant approach such as On-premises. The longer-term total cost of on-premises ownership was also compared with the increasingly popular cloud subscription model. Comparison results show that the On-Premises cost structure will likely be more efficient over the long term. According to Cong et al. [52], perceived costs typically consist of money, time, and labor. Often, perceived cost includes both monetary and non-monetary costs (i.e., service price and service waiting time, respectively). Since cloud subscriptions could be more expensive over the years, this could significantly affect consumers' choices. Thus, in this study, it was hypothesized that.

Hypothesis **(PC → BI)**: PC negatively affects BI to use CS services.

#### 2.5.3 Perceived risk

Perceived Risk (PR) constitutes a significant barrier to accepting or using new technologies. When deciding whether or not to adopt a particular technology, consumers weigh the PR against the technology's convenience. PR can significantly impact users' adoption decisions [53]. Risks in cloud computing are mainly about security and privacy concerns [54]. There is a significant increase in cloud security threats as new technologies emerge to meet user demands. These threats manifest as multiple unexpected exploits of cloud computing services and their associated interfaces. Preventing attacks in the future and minimizing their potential impacts are critical objectives that must be addressed. The presence of vulnerable interfaces is a significant obstacle for cloud consumers and CS service providers as well [55]. The issues mentioned above impede cloud computing adoption. Thus, it was hypothesized that.

Hypothesis **(PR → BI)**: PR negatively affects BI to use CS services.

### 2.6 Proposed conceptual framework

**Fig. 1:**
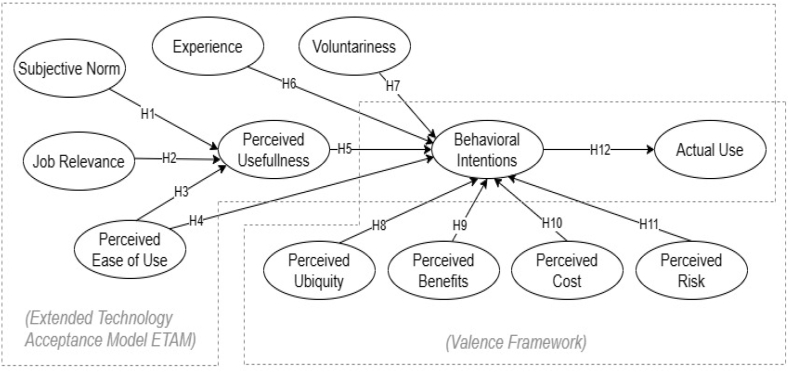
Proposed conceptual model.

The hypotheses were developed using the ETAM and the Valance Framework of Behavioral Beliefs discussed in this chapter. These hypotheses are the basis of the proposed model. The hypotheses were labeled H1 to H12 as depicted in Fig. 1. Additionally, all generated hypotheses that this study aimed to explore are detailed in Table 2.

**Table 2:** Summary of generated hypotheses.

Model	Hypothesis	Label	Detail	Reference
ETAM	(SN → PU)	H1	SN influences PU.	[42,43]
	(JR → PU)	H2	JR influences PU.	[45,46]
	(PEOU → PU)	H3	PEOU positively influences PU	[40]
	(PEOU → BI)	H4	PEOU positively influences BI to use CS services	[40]
	(PU → BI)	H5	PU positively influences BI to use CS services.	[40]
	(EX → BI)	H6	EX positively affects BI to use CS.	[44]
	(VO → BI)	H7	VO positively affects BI to use CS.	[44]
	(BI → AU)	H12	BI positively influences Actual Use.	[39]
VALENCE FRAMEWORK	(PQ → BI)	H8	PQ positively affects BI to use CS services.	[36]
	(PB → BI)	H9	PB positively influence BI to use CS services.	[36]
	(PC → BI)	H10	PC negatively affects BI to use CS services	[[49], [50], [51], [52]]
	(PR → BI)	H11	PR negatively affects BI to use CS services.	[[53], [54], [55]]

## 3 Methodology

### 3.1 Survey design and Questionnaire

The survey consists of two parts. The first part was the demographic information, which contained questions to build descriptive statistics about the respondents. Additionally, because the survey's latter part is geared toward CS users, the first part is crucial in determining who uses CS among the respondents. Finally, the last part included questions about the generated hypotheses, detailed in Table 2. These questions were anticipated to reveal the factors affecting Filipino consumers' behavioral intention to use CS services. A comprehensive set of 52 questions derived from several studies, encompassed all relevant constructs. All the questions in English were the medium utilized for this study, presented in the supplementary file are the measure items gauged using a 7-point Likert scale. Before deployment, this study was approved by a university ethics committee (FM-RC-23-01-45).

### 3.2 Data collection

For this study, data is gathered through an online survey conducted on Google Forms and disseminated through various online communication platforms such as Viber, LinkedIn, Facebook and its groups, and WhatsApp to determine factors affecting Filipino consumers' behavioral intention to use CS services. A minimum of 400 participants is needed for this study to increase the reliability and generalizability of the survey. The number of target participants is based on the Yamane Taro Formula (eq. (1)), wherein n = sampling size; e = sampling error equal to 5%; and N = population size and equal to 69,397,349 Filipinos aged 15 to 64 according to the 2020 Census of Population and Housing Summary of Philippine Statistics Authority [[56], [57], [58]].

(eq. 1) $$n=\frac{N}{1+N\left(e^{2}\right)}$$

Convenience sampling, which was utilized for data gathering, is a common type of sampling used in population research because it is inexpensive, less time-consuming than other sampling strategies, and straightforward. In convenience sampling, the researcher announces the study, recruits the most accessible respondents, and then the participants decide to participate. Convenience sampling is beneficial to generate a possible hypothesis or study objective [59].

The survey ran from November 1, 2022, to February 28, 2023, and was open to all Filipinos residing in the Philippines. In Table 3, responses from 467 participants were analyzed and narrowed down to 431 participants identified as Cloud Users (consent collected FM-RC-23-02-45). Table 4 shows that respondents are mainly employed individuals, residing in Region IV-A (Calabarzon) and NCR (National Capital Region), which prefer Google Drive for CS.

**Table 3:** Types of respondents, N = 467.

Characteristics	Category	N	%
I am using/I have experienced using CS services.	Yes	431	92.3%
	No	36	7.7%

**Table 4:** Demographic Profile of CS users (n = 431).

Characteristics	Category	N	%
Gender	Female	177	41.1%
	Male	242	56.1%
	Prefer not to say	12	2.8%
Age	15–28	109	25.3%
	29–38	201	46.6%
	39–48	105	24.4%
	49–58	14	3.2%
	59–64	2	0.5%
Educational Level	High school	11	2.6%
	College	373	86.5%
	Master's Degree	40	9.3%
	PhD	1	0.2%
	Other	6	1.4%
Type of University/School	Private	191	44.3%
	Semi-Private	49	11.4%
	Public	188	43.6%
	Other	3	0.7%
Access to Internet	Weak	24	5.6%
	Moderate	263	61.0%
	Strong	144	33.4%
Monthly Salary/Allowance	Less than ₱15,000	32	7.4%
	₱15,000 - ₱30,000	159	36.9%
	₱30,000 - ₱45,000	146	33.9%
	₱45,000 - ₱60,000	37	8.6%
	₱60,000 - ₱75,000	20	4.6%
	More than ₱75,000	37	8.6%
Location	Region I (Ilocos Region)	4	0.9%
	Region II (Cagayan Valley)	3	0.7%
	Region III (Central Luzon)	8	1.9%
	Region IV-A (Calabarzon)	134	31.1%
	Region IV-B (Mimaropa)	1	0.2%
	Region V (Bicol Region)	0	0%
	CAR (Cordillera Administrative Region)	1	0.2%
	NCR (National Capital Region)	280	64.5%
	Region VI (Western Visayas)	2	0.5%
	Region VII (Central Visayas)	0	0%
	Region VIII (Eastern Visayas)	0	0%
	Region IX (Zamboanga Peninsula)	0	0%
	Region X (Northern Mindanao)	0	0%
	Region XI (Davao Region)	0	0%
	Region XII (Soccsksargen)	0	0%
	Region XIII (Caraga)	0	0%
	BARMM (Bangsamoro)	0	0%
Nature of Work	Agriculture, forestry, and fishing	0	0%
	Mining and quarrying	2	0.5%
	Manufacturing Industry	86	20%
	Electricity, gas, steam and air-conditioning Supply	0	0%
	Water supply, sewerage, waste-management and remediation activities	0	0%
	Construction	4	0.9%
	Wholesale and retail trade; repair of motor vehicles and motorcycles	4	0.9%
	Transportation and storage	2	0.5%
	Accommodation and food service activities	0	0%
	Information and communication	15	3.5%
	Educational Services	9	2.1%
	Financial and insurance activities	11	2.6%
	Healthcare and Social Assistance	5	1.2%
	Real estate activities	7	1.6%
	Professional, scientific, and technical Services	42	9.7%
	Administrative and support service Activities	25	5.8%
	Arts, entertainment and recreation	0	0%
	Activities of extraterritorial organizations and bodies	2	0.5%
	Activities of private households as employers and undifferentiated goods and services and producing activities of household for own use.	0	0%
	Student	112	25.95%
	Other	105	24.35%
I use/have used one or more of the following CS Services: Google Drive; OneDrive; Dropbox; Amazon Cloud Drive; Apple iCloud	Apple iCloud	9	2.1%
	Google Drive	169	39.2%
	Google Drive and Amazon Cloud Drive	2	0.5%
	Google Drive and Apple iCloud	30	7.0%
	Google Drive and Dropbox	10	2.3%
	Google Drive and OneDrive	57	13.2%
	Google Drive and Other more	154	35.7%
		

From the collected data, the Shapiro-Wilk test for normality was conducted. Results presented that the quotient of the skewness and kurtosis are within ±1.96 as evident in Table 5. This indicates an acceptable normality distribution of data collected [60]. In addition, the run for common method bias using Harman's Single Factor Test presented 23.78% which indicates no common method bias (<25%) [61].

**Table 5:** Shapiro-Wilk test.

Items	Kurtosis	Skewness	SWT
SN1	−1.427	0.059	−0.04130
SN2	−1.431	−0.143	0.09993
SN3	−1.308	0.118	−0.0902
SN4	−1.396	−0.069	0.04943
SN5	−1.303	−0.307	0.23561
JR1	−0.777	−0.759	0.97683
JR2	−0.631	−0.824	1.30586
JR3	−0.624	−0.790	1.26603
JR4	−0.640	−0.777	1.21406
JR5	−0.498	−0.872	1.75100
PU1	−0.519	−0.777	1.49711
PU2	−0.468	−0.807	1.72436
PU3	−0.289	−0.228	0.78893
PU4	−0.299	−0.328	1.09699
PU5	−0.300	−0.317	1.05667
PU6	−0.401	−0.368	0.91771
PEOU1	−0.357	−0.291	0.81513
PEOU2	−0.344	−0.209	0.60756
PEOU3	−0.423	−0.768	1.81560
PEOU4	−0.341	−0.634	1.85924
EX1	−0.867	−0.561	0.64706
EX2	−0.529	−0.749	1.41588
EX3	−0.711	−0.524	0.73699
VO1	−0.714	−0.699	0.97899
VO2	−1.231	−0.140	0.11373
VO3	−1.206	−0.050	0.04146
VO4	−1.244	−0.035	0.02814
PQ1	−0.716	−0.695	0.97067
PQ2	−0.503	−0.791	1.57256
PQ3	−0.551	−0.761	1.38113
PQ4	−0.519	−0.717	1.38150
PB1	−0.611	−0.563	0.92144
PB2	−0.484	−0.644	1.33058
PB3	−0.430	−0.754	1.75349
PB4	−0.698	−0.501	0.71777
PR1	−0.837	−0.245	0.29271
PR2	−0.837	−0.394	0.47073
PR3	−0.898	−0.150	0.16704
PR4	−0.773	−0.513	0.66365
PR5	−0.751	−0.415	0.55260
PR6	−0.807	−0.417	0.51673
PC1	−0.987	−0.162	0.16413
PC2	−0.980	−0.143	0.14592
PC3	−0.992	−0.188	0.18952
PC4	−1.006	−0.041	0.04076
BI1	−0.534	−0.701	1.31273
BI2	−0.458	−0.705	1.53930
BI3	−0.496	−0.645	1.30040
BI4	−0.633	−0.467	0.73776
AU1	−0.953	−0.475	0.49843
AU2	−0.985	−0.396	0.40203
AU3	−0.937	−0.475	0.50694

### 3.3 Analysis Procedure

Structural Equation Modelling (SEM) helps determine how variables affect each other; evaluated through the causal relationship present in the objective framework considered. SEM lets researchers examine how predictor variables affect several dependent variables at once. Moreover, SEM provides accounting for measurement errors and predicts relationship errors, making it suitable for simultaneous analysis of extended frameworks [60,62]. Furthermore, SEM can evaluate a full model instead of merely focusing on individual relationships [62].

As explained by Dash and Paul [63], the utilization of SEM analysis could provide insight into relationships evaluating social science and technology acceptance models. Either the PLS-SEM or the covariance-based SEM (CB-SEM) could be used for analysis. The only difference highlighted that PLS-SEM is better suited for analyzing newly developed models since it is sensitive. However, since this study considered an established model and simple integration, CB-SEM would suffice the analysis. In accordance, the study of Prasetyo et al. [64] considered CB-SEM to analyze their study on e-learning platform acceptance with the integrated TAM and Delone and McLean IS Model. From their results, a positive reflection of constructs and path analyses was obtained.

In accordance, the study of Rosli et al. [65] also highlighted the use of CB-SEM in their study covering TAM review works of literature and indicated that CB-SEM has been proven to be effective in analyzing technology acceptance. Thus, this research uses SEM alongside the AMOS22 and SPSS25 programs. AMOS is an SPSS-integrated program with a graphical user interface. Other data entry programs are available, but SPSS is the most user-friendly of all SEM software programs [66,67].

## 4 Results, analysis and discussion

### 4.1 Initial and final SEM analysis

**Fig. 2:**
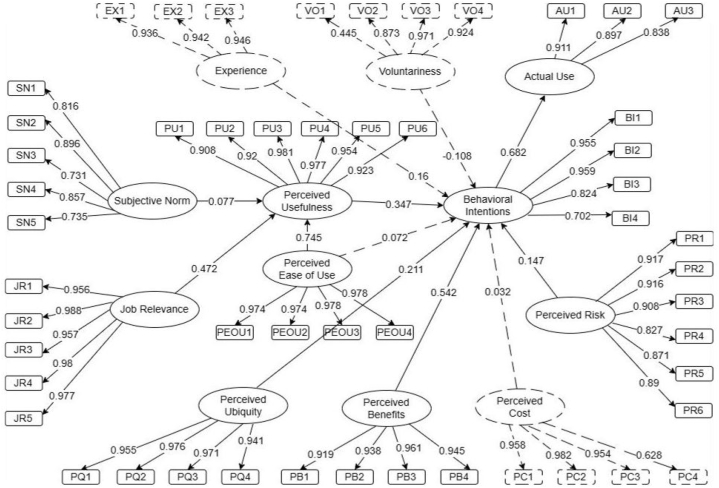
Initial structural model.

Fig. 2 depicts the adapted Amos Graphics Software model developed based on the proposed conceptual framework, which was utilized in the initial SEM analysis. The ellipses in Fig. 2 represent latent variables that constitute the factors affecting Filipino consumers' behavioral intentions to use CS services. In contrast, the rectangles are the observed variables, which also serve as the indicators that evaluate each factor. The single-headed arrows represent the regression coefficients. These arrows demonstrate the causal relationship between items and are also known as factor loading [68,69].

In the initial SEM analysis, the factor loading value of each item in the observed variables and the p-value of each relationship between variables were examined to determine whether they were significant. It is considered significant if each item's factor loading value exceeds 0.5 and the p-value of each relationship is less than 0.05. Fig. 2 shows that one of the items, VO1, has low factor loading and is therefore considered insignificant. Further checking on the p-values in Table 6 shows that four out of the twelve established relationships have high p-values, thus regarded as insignificant [60,64,67].

**Table 6:** Standardized direct effects from the initial SEM analysis.

	Hypothesis	Relationship	p-value	Remarks
1	H3	PEOU→PU	0.030	Significant
2	H2	JR→PU	0.003	Significant
3	H1	SN→PU	0.044	Significant
4	H11	PR→BI	0.007	Significant
5	H4	PEOU→BI	0.645	Not Significant
6	H10	PC→BI	0.552	Not Significant
7	H9	PB→BI	0.009	Significant
8	H8	PQ→BI	0.014	Significant
9	H7	VO→BI	0.099	Not Significant
10	H6	EX→BI	0.105	Not Significant
11	H5	PU→BI	0.004	Significant
12	H12	BI→AU	0.041	Significant

**Fig. 3:**
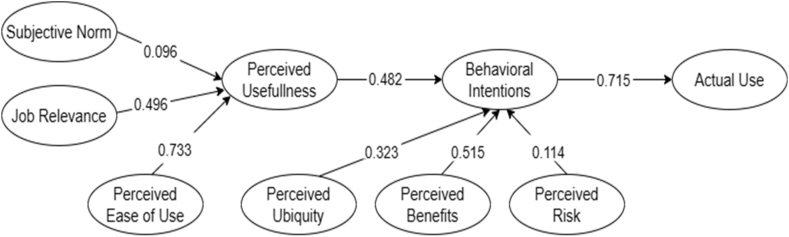
Final structural model.

Modification indices were also utilized to improve the model fit further. Furthermore, all insignificant components in the model were removed for an acceptable model fit. Therefore, in Fig. 3, it can be seen that the relationship between PEOU→BI and the latent variables EX, VO, and PC has been eliminated in the final SEM analysis for this study.

### 4.2 Confirmatory Factor Analysis

Confirmatory Factor Analysis (CFA) is a part of SEM. CFA is a theory or model-driven approach that examines how well data "fits" the proposed model or theory. CFA employs maximum likelihood estimation (MLE) and various standards to evaluate the suitability of the construct of interest [67,68]. In CFA, factor loading represents the correlation between items and the factor. The factor loading value should be greater than 0.50 to demonstrate statistical significance [70]. Table 6 shows that all final factor loading exceeded the cut-off value, indicating that the remaining latent variables are significant.

In addition to the factor loading, Table 7 also displays the reliability and validity of the constructs, including Cronbach's alpha, composite reliability (CR), and Average Variance Extracted (AVE). Cronbach's alpha reliability ranges from 0 to 1, and the minimum level of internal reliability should be higher than 0.70. A higher reliability value implies strong internal consistency between the items [67]. While a CR value of at least 0.70 is required to demonstrate acceptable reliability and convergent validity levels. Convergent validity indicates the extent to which the measures of the constructs are related to each other [71]. Finally, the AVE evaluates the discriminant validity of the constructs. The measurements were accurate, and the convergent validity test was passed if the AVE was higher than 0.50. In summary, internal consistency measures such as Cronbach's alpha and CR must be greater than 0.700, while AVE must be more than 0.50 for each item set in a construct. All constructs exceeded the minimum required values for the AVE, Cronbach alpha, and CR, which indicates that this study's reliability and validity tests were deemed "Passed” [40,60].

**Table 7:** Results of CFA and Reliability/Validity test.

Variable	Item	Mean	Std. Dev.	Load Factor		Reliability and Validity		
				Initial SEM	Final SEM	Cronbach's α	Composite Reliability (CR)	Average Variance Extracted (AVE)
Subjective Norms	SN1	3.889	1.96	0.816	0.852			
	SN2	4.22	1.995	0.896	0.892			
	SN3	3.784	1.883	0.731	0.687	0.903	0.903	0.654
	SN4	4.153	1.953	0.857	0.858			
	SN5	4.585	1.966	0.735	0.735			
Job Relevance	JR1	5.121	1.947	0.956	0.956			
	JR2	5.2	1.884	0.988	0.989			
	JR3	5.088	1.851	0.957	0.957	0.988	0.988	0.945
	JR4	5.139	1.825	0.98	0.98			
	JR5	5.232	1.874	0.977	0.977			
Experience	EX1	4.968	1.744	0.936	–			
	EX2	5.146	1.658	0.942	–	–	–	–
	EX3	4.916	1.594	0.946	–			
Voluntariness	VO1	5.019	1.8	0.445	–	–	–	–
	VO2	4.227	1.845	0.873	–			
	VO3	4.049	1.836	0.971	–			
	VO4	3.947	1.876	0.924	–			
Perceived Usefulness	PU1	5.056	1.724	0.908	0.918	0.988	0.979	0.888
	PU2	5.012	1.726	0.92	0.923			
	PU3	5.213	1.766	0.981	0.971			
	PU4	5.218	1.77	0.977	0.959			
	PU5	5.227	1.741	0.954	0.957			
	PU6	5.107	1.743	0.923	0.925			
Perceived Ease of Use	PEOU1	5.111	1.675	0.974	0.974	0.988	0.988	0.953
	PEOU2	5.077	1.644	0.974	0.974			
	PEOU3	5.056	1.674	0.978	0.978			
	PEOU4	5.179	1.678	0.978	0.978			
Perceived Ubiquity	PQ1	4.845	1.784	0.955	0.955	0.979	0.98	0.923
	PQ2	5.039	1.742	0.976	0.976			
	PQ3	4.954	1.766	0.971	0.971			
	PQ4	4.868	1.753	0.941	0.941			
Perceived Benefits	PB1	4.91	1.706	0.919	0.911	0.969	0.97	0.889
	PB2	4.968	1.669	0.938	0.952			
	PB3	5.158	1.73	0.961	0.956			
	PB4	4.956	1.686	0.945	0.952			
Perceived Cost	PC1	4.037	1.667	0.958	–	–	–	–
	PC2	4.09	1.663	0.982	–			
	PC3	4.088	1.704	0.954	–			
	PC4	3.949	1.647	0.628	–			
Perceived Risk	PR1	4.234	1.648	0.917	0.816	0.957	0.951	0.767
	PR2	4.362	1.651	0.916	0.863			
	PR3	4.132	1.609	0.908	0.798			
	PR4	4.71	1.78	0.827	0.878			
	PR5	4.492	1.679	0.871	0.961			
	PR6	4.503	1.717	0.89	0.926			
Behavioral Intention	BI1	5.009	1.666	0.955	0.911	0.965	0.905	0.706
	BI2	5.067	1.659	0.959	0.909			
	BI3	4.968	1.692	0.824	0.822			
	BI4	4.689	1.643	0.702	0.702			
Actual Use	AU1	4.698	1.802	0.911	0.966	0.946	0.893	0.737
	AU2	4.517	1.779	0.897	0.836			
	AU3	4.589	1.762	0.838	0.761			

### 4.3 Causal relationship between latent variables

Table 8 shows the coefficient β of the standardized effect (direct, indirect, and total effects) between the latent variables' causal relationships. The idea of "direct effect" refers to quantifying an influence not mediated by other variables within the model. A variable has a direct effect on another variable if it is not intervened by another variable [72]. Consequently, a variable has an indirect effect on another variable when the effect is exerted by at least one mediating variable. Notably, under the standardized direct effect, PEOU towards PU has the highest value (β = 0.733). In contrast, PB towards AU appears to have the highest standardized indirect effect with β of 0.368.

**Table 8:** Standardized direct, indirect, and total effects from the final SEM analysis.

Causal Relationship between latent variables				Direct Effect		Indirect Effect		Total Effect	
				β	p-value	β	p-value	β	p-value
1	SN	→	PU	0.096	0.016	–	–	0.096	0.016
2	JR	→	PU	0.496	0.003	–	–	0.496	0.003
3	PEOU	→	PU	0.733	0.025	–	–	0.733	0.025
4	PR	→	BI	0.114	0.031	–	–	0.114	0.031
5	PEOU	→	BI	–	–	0.353	0.003	0.353	0.003
6	PB	→	BI	0.515	0.009	–	–	0.515	0.009
7	PQ	→	BI	0.323	0.014	–	–	0.323	0.014
8	JR	→	BI	–	–	0.239	0.002	0.239	0.002
9	SN	→	BI	–	–	0.047	0.01	0.047	0.01
10	PU	→	BI	0.482	0.005	–	–	0.482	0.005
11	PR	→	AU	–	–	0.081	0.027	0.081	0.027
12	PEOU	→	AU	–	–	0.253	0.004	0.253	0.004
13	PB	→	AU	–	–	0.368	0.012	0.368	0.012
14	PQ	→	AU	–	–	0.231	0.018	0.231	0.018
15	JR	→	AU	–	–	0.171	0.003	0.171	0.003
16	SN	→	AU	–	–	0.033	0.011	0.033	0.011
17	PU	→	AU	–	–	0.345	0.01	0.345	0.01
18	BI	→	AU	0.715	0.03	–	–	0.715	0.03

### 4.4 Hypothesis testing

Table 8 displays the eight remaining hypotheses and their respective p-values from the final SEM analysis. All p-values are less than 0.05, indicating significant causal relationships between latent variables. In addition, the p-values validate and support the hypotheses presented in Table 9.

**Table 9:** Validated and supported hypotheses in the final SEM analysis.

Hypotheses		Relationship	Β	p-value	Remarks
**ETAM**	H1: Subjective Norm (SN) influences PU.	SN → PU	0.096	0.016	Supported
	H2: Job Relevance (JR) influences PU.	JR → PU	0.496	0.003	Supported
	H3: Perceived Ease of Use (PEOU) positively influences PU	PEOU → PU	0.733	0.025	Supported
	H5: Perceived Usefulness (PU) positively influences BI to use CS services.	PU → BI	0.482	0.005	Supported
	H12: Behavioral Intension (BI) positively influences Actual Use.	BI → AU	0.715	0.03	Supported
**VALENCE** **FRAMEWORK**	H8: Perceived Ubiquity (PQ) positively influences BI to use CS services.	PQ → BI	0.323	0.014	Supported
	H9: Perceived Benefits (PB) positively influence BI to use CS services.	PB → BI	0.515	0.009	Supported
	H11: Perceived Risks (PR) negatively influences BI to use CS services.	PR → BI	0.114	0.031	Supported

### 4.5 Model fit

Model fit refers to how well the hypothesized model explains the data. Using the fit indices, researchers can determine how well their structural equation models correspond with their data [73]. Several indices are available to assess model fit, including the incremental fit index (IFI), which incorporates the Tucker-Lewis index (TLI), the comparative fit index (CFI), the goodness of fit index (GFI), the adjusted goodness of fit index (AGFI), and the root mean square error (RMSEA). IFI constitutes an essential class of fit indices that expresses the fit of a substantive model as falling somewhere between the fit of a highly restrictive “null” model and that of a saturated (perfectly fitting) model [74].

TLI quantifies the extent to which misfit is reduced concerning the degrees of freedom. TLI was first introduced within the framework of exploratory factor analysis and then extended to the context of covariance structure analysis. CFI assesses the adequacy of a model by comparing its fit to that of a null or independent model. One notable distinction lies in its focus on hidden components as opposed to indicators. GFI serves as an alternative to the chi-square test that estimates the proportion of the variance provided by the projected covariance of the population. AGFI is a statistical measure derived from GFI that tries to adjust the GFI with degrees of freedom. For example, mean squares are used instead of the sum of squares (used in GFI). Finally, RMSEA is widely regarded as a highly useful fit index. The objective is to determine the ideal number of parameters, namely a smaller number, that may effectively capture the characteristics of the final population covariance matrix [63,71,75]. In Table 10, except for the RMSEA, which must be less than 0.07 [76], all other indices mentioned above must be greater than 0.8 [77] to indicate a good model fit. Based on the obtained data, it can be inferred that all of the fit values generated for this study satisfy the specified criteria, suggesting a "Good fit" for the model.

**Table 10:** Model fit.

Goodness of fit measures of SEM	Parameter Estimates	Min Cut-Off	Reference
Incremental Fit Index (IFI)	0.971	>0.80	[77]
Tucker Lewis Index (TLI)	0.958	>0.80	[77]
Comparative Fit Index (CFI)	0.936	>0.80	[77]
Goodness of Fit Index (GFI)	0.832	>0.80	[77]
Adjusted Goodness of Fit Index (AGFI)	0.854	>0.80	[77]
Root Mean Square Error (RMSEA)	0.047	<0.07	[76]

### 4.6 Discussion of results

This study integrated two theoretical framework models, the ETAM and the Valence Framework, and utilized SEM for the analysis. This study aimed to determine the factors affecting Filipino consumers' behavioral intention to use CS services. This study evaluated the factors SN, JR, PU, PEOU, EX, VO, PQ, PB, PR, PC, BI, and AU. Filipinos from different walks of life participated in the online survey from November 1, 2022, to February 28, 2023, and the results are as follows.

As shown in Table 8, PEOU towards PU (H3, β = 0.733, p = 0.025) has the strongest direct relationship among hypotheses. It has been found that CS services are easy to use and that learning how to use these is simple and convenient since they are accessible even via mobile devices. PEOU refers to an individual's perception that using a technology requires little effort. In other words, users of new technology can do particular work quite easily when comparing the required work and perceived benefits among Vietnamese [78]. However, PEOU shows no direct effect on BI in this study (H4, PEOU → BI). PEOU only has an indirect effect on BI (β = 0.353, p = 0.003) through PU. This result is in line with Cengiz and Bakirtas from Turkey [79], wherein findings that the BIs and use of technology were influenced directly by PU and indirectly by PEOU. Interestingly, they also concluded that the PEOU of cloud computing technology was not influenced by anxiety, playfulness, or perceived enjoyment. The most influential factor in PEOU is self-efficacy. Self-efficacy is regarded as a significant factor influencing users' decisions on utilizing computer-related technology, and it is an essential factor in the continued use of cloud computing services. Therefore, it is confirmed that PEOU is a strong determinant of PU, which indirectly influences consumers' BI to use CS services. CS providers must ensure that their products and services are more user-friendly by keeping their systems as straightforward as possible so that consumers can smoothly execute their actions to achieve particular performance outcomes.

JR and SN are additional latent variables that directly affect PU, and similar to PEOU, these also indirectly affect BI. In the case of JR towards PU (H2, β = 0.353, p = 0.003). JR refers to a user's perception of how much a particular system or technology corresponds with their job responsibilities. The relevance of a system or technology to a job is determined by the value of the set of activities it can support within a given position [45,46]. Consumers may compare what a system or technology can do with what they have to accomplish in their jobs to determine if a system or technology would be useful. This was evident in both Saudi Arabia and Sri Lanka. In this study, it is evident from the findings that CS services are relevant to the consumers' jobs in storing and accessing work-related files or information. Stored data would always be readily available for analysis and reporting purposes. In addition, data can be accessed at any time and remain accessible even without the data owner. The statements mentioned above indicate that CS services can support the duties of a working individual or an employee. With this, JR is inferred to predict PU. CS providers should tailor their services to their target industries' job or task requirements.

As illustrated in the conceptual framework, all latent variables are anchored to BI. Moreover, BI is the only latent variable directly influencing AU. In Table 8, only BI was observed with a direct effect coefficient toward AU (H12, β = 0.715, p = 0.03), while all other latent variables only have an indirect effect coefficient on AU. Analyzing all factors that directly contributed to BI, it is evident that PB (H9, β = 0.515, p = 0.009) and PU (H5, β = 0.482, p = 0.005) towards BI are the most critical factors. Notably, latent variables with the most significant indirect effect on AU are still PB (β = 0.368) and PU (β = 0.345). In contrast, PR has the lowest indirect effect coefficient (β = 0.081). This means BI strongly correlates with consumers' favorable perceptions of CS services, leading to actual utilization. Consumers will likely use the technology due to its usefulness and benefits in archiving their work and personal data.

The positive functionality feature of the valence framework assesses the extent to which adopting services can provide users with relative gains. PB is one of the positive features of the valence framework in this study. It has been seen that PB towards BI is significant since users benefit from not having to worry about the space and maintenance a server typically requires. Users do not have to worry about data loss because multiple backups are deployed globally. Also, users do not need to purchase security software because CS providers already run it to protect clients' private information. Business enterprises also enjoy many benefits from cloud computing, leading to a shift in their perception of the system primarily because of its maintenance-free environment. In summary, cost reduction, scalability, portability, as well as reduced software and hardware obsolescence are some of the perceived benefits of cloud computing [17,36,80].

PU is grounded in the notion that utilizing a service would yield positive outcomes. PU significantly influences BI because it measures users' subjective assessments of the usefulness in terms of specific related goals such as productivity and performance [81]. In this study, PU towards BI is found to be significant in terms of faster archiving and retrieving personal and work-related files. Consumers view this as applicable because it enhances their efficiency and productivity. Moreover, CS services simplify consumers' work by providing more extensive data storage and as a convenient and sophisticated means of file sharing. For instance, instead of attaching large files to emails or passing along physical storage devices such as thumb drives, users can send their colleagues links to work-related files that can be accessed anytime. The cloud enables organizations to administer large amounts of data with a single click and improves industry accessibility. Cloud technology is one method for organizations to manage all of their data. Some companies use shared computing services to reduce their computing costs and store data on a server like that in Pakistan [82]. As PU substantially impacts an individual's or organization's BI, CS providers could emphasize the value proposition of cloud archiving to increase the technology's adoption.

Since PU and PB were found in this study to have a significant direct effect on BI and an indirect effect on AU, CS providers should offer quality services to be considered valuable and beneficial to consumers, such as faster data access time. Also, the user should never receive a denial of service, regardless of the condition. Even for the worst natural disasters, a cloud provider should have all contingency plans in place; as seen from the study in India [83]. In addition, cloud providers from could make their infrastructure eco-friendlier by incorporating energy-efficient devices and components that could help reduce their carbon footprint [84]. When environmentally conscious consumers learn that CS also provides environmental benefits, they will be more likely to adopt CS technology.

PQ is also a positive feature of the valence framework, and it was found that PQ towards BI (H8, β = 0.323, p = 0.014) is significant in terms of ubiquitous access. Even mobile devices, provided with the internet, can access CS services from any location and anytime. Users do not need to be concerned about whether they can access their files while away from their homes or workplaces. There are no geographical restrictions on cloud computing's accessibility, so it is accessible anywhere and anytime. It helps users overcome the physical challenges of carrying devices and interfaces to bring their data. Therefore, given the omnipresent characteristics of cloud computing such as that in India, perceived ubiquity is confirmed as a positive determinant of behavioral intention to adopt the technology [85].

According to a study from Jordan by Atobishi et al. [54], the primary concerns associated with cloud computing are its security and privacy risks. Data security is the main impediment in selecting cloud computing, accompanied by many other substantial challenges, such as privacy, trust, legal issues, and compliance. Their study identified several security and privacy concerns in the cloud computing environment, including accountability, confidentiality, privacy preservation in the event of a loss of physical control over data, integrity due to data manipulation or dishonest calculation on remote servers, and bandwidth and pricing availability issues. Their findings concluded that PR is statistically significant and has a direct negative impact on the BI to use. Similar to their findings, PR was also found to directly affect BI (H11, β = 0.114, p = 0.031) in this study. Users are possibly specifically concerned about the associated risk with providing personal and banking information during CS payment transactions since PR5 and PR6 were observed to have the highest loading factors among the indicators of PR.

According to Gupta and Kumar from India [83], the most challenging aspect of cloud computing is maintaining data security and confidentiality. Protecting and securing personal and sensitive data stored in the cloud is paramount. The data must be for private use only. Users store information ranging from the least private to the most private in the cloud. Since the user places sensitive data on his cloud account, the CS provider must ensure that the data is secure in the best way possible. This challenge can be mitigated with encrypted file systems and data loss prevention software. CS service providers must also pay attention and actively shape their reputation as reliable storage providers to boost users' BI to use the technology. This can be achieved by continuously informing consumers about how their sensitive data is being protected [55].

The relationship between SN and PU (H1, β = 0.096, p = 0.016) is also significant, but it is the least significant among the hypotheses. Further evaluating SN's factor loading values in this study revealed that SN2 has the highest value among indicators. Since most respondents are employed individuals, it could imply that users receive job-related insights from their colleagues or peers. Therefore, it can be deduced that SN moderately impacted PU based on the results of this study. This study's outcome is comparable to that of Santoso et al. [43], who confirmed that SN exhibits a considerable influence on BI. Their findings indicated that a friend or family member can influence a user's interest in storing data in the cloud. Additionally, it can also be inferred that the adoption of CS technology in developing countries (*e.g.,* Malaysia) is influenced by group opinions rather than personal beliefs [85,86].

Insignificant factors in this study are the PC, EX, and VO. The PC is one of the negative utilities of the valence framework associated with the monetary expense of employing cloud services. Numerous CS providers offer a portion of their storage space for free. Thus, users may only utilize storage space up to the free limit. In addition, the survey includes questions about financial loss if users' data are lost or compromised. The data participants store in their cloud accounts may not be sensitive or confidential. As a result, they will incur no financial loss if their data are lost or compromised. However, the assumptions mentioned above cannot be confirmed because the survey did not inquire about the type of data respondents store or the average amount of storage space the users usually consume.

This study also found that EX was an insignificant variable, and this finding is identical to that of Purnama and Ginardi's [87] research on a cloud computing application used in Indonesian banking with TAM2. Their research revealed that EX was not a significant factor and concluded that prior experience with a similar app is unnecessary to use the new app under study. Furthermore, many bank employees remained interested in using the new application because they believed it would benefit their work. Therefore, participants in this study employed CS not due to their prior exposure to the technology but rather due to their perception that CS provides several benefits compared to traditional storage devices.

As for the VO, it could be insignificant since the survey primarily inquired whether participants' bosses and instructors required them to use CS for their work duties or school activities. The survey did not include questions regarding the potential necessity of CS for their work or school commitments. Participants are compelled to use CS if their commitments necessitate CS, regardless of explicit instructions.

It could also be possible that the questions in the survey confused the participants, just like in the study of Schuster et al. [88] from Germany, where participants were asked first if they voluntarily participated in their study, to which they replied, "Yes." After that, participants were asked once more if they voluntarily used the system that had been introduced to them, even though the study design required the said system. In this study, the participants were asked if their superiors at work or school instructors required them to use CS. Following that, participants were asked again if the organizations they belong to do not require them to use CS, but their work or school commitments could necessitate the usage of CS.

In summary, the findings indicated that H3 (PEOU→PU) has the strongest direct relationship among the twelve hypotheses; however, PEOU was found to have no direct effect on the BI. Comparing all the factors that directly contributed to the BI, the PB and the PU emerged as the most significant. While the PC, EX, and VO were revealed to be insignificant factors in this study. It could be posited that the aforementioned findings suggest that consumers consider a technology to be useful not only if it can be used, but also if it can be utilized effortlessly. Although consumers may perceive a technology to be useful if it is easy to use, this does not automatically translate into actual usage of the technology. Furthermore, when consumers find a technology not just useful but also beneficial to their activities or lifestyle, this will influence their behavioral intention and eventually lead to actual usage of the technology.

Voluntariness was found to be one of the insignificant factors which indicates that the environment in which consumers find themselves does not influence their intention to utilize the technology. The experience was also found not a significant factor and this means that although exposure to the technology can heighten consumers' awareness of the technology, this does not have an impact on their intentions to use the technology. Interestingly, the perceived cost was also deemed insignificant, probably due to the availability of free, although with certain limitations, storage options provided by CS providers. Lastly, while the perceived cost and experience were also found to be not significant to consumers’ behavioral intention, it would still be best if technology is made more affordable, not complicated, and would not require prior knowledge or experience with comparable technologies to be able to use it.

### 4.7 Theoretical Contribution

Based on the publications in the past decade, TAM is commonly employed in the following applications, listed in order of popularity: electronic commerce/e-commerce, internet banking/online banking, social media/social networks, e-learning, e-government, mobile commerce/m-commerce, mobile learning (m-learning), mobile banking, cloud computing, and augmented reality. While the top ten countries with the highest TAM publications are China, the USA, Taiwan, Malaysia, South Korea, Spain, England, Indonesia, Australia, and India [14], the Philippines is among the countries that utilize cloud storage [8,9] but has yet to have holistic assessment aside from the current study.

This information indicates that while TAM is employed in diverse applications and widely accepted as a research model globally, Cloud Computing is not as prevalent a field as e-commerce. Additionally, the predominantly developed countries tend to have a greater number of research studies utilizing TAM. Hence, this study adds to the little literature that integrated Valence frameworks into the ETAM and utilized SEM to analyze consumer adoption of CS services in a developing nation, particularly the Philippines.

Furthermore, the two theoretical frameworks were combined to develop a comprehensive model for this study. In most literature, EX and VO give mediating effects as these are connected between the SN and BI. In this research, the conceptual model is different since EX and VO are directly connected to the BI. Unfortunately, EX and VO did not yield statistically significant results. Nevertheless, this research still adds to the literature on ETAM, where EX and VO do not have mediating effects because these are directly anchored to the BI. Lastly, while this study focuses on CS services, it can also be used as a reference for further studies on how consumers adopt new technologies being used in various industries, such as e-commerce, healthcare, sports and leisure, education, and more.

### 4.8 Practical implication

CS providers could use the findings of this study as a guide for improving their services. Based on the SEM results, PB and PU significantly influenced BI and AU of technology usage. Therefore, CS providers should prioritize finding additional ways to make their offerings more beneficial to consumers, such as faster access time and cheaper but higher storage capacities. Moreover, since perceived risk and subjective norm were also found to be significant in this study, CS providers should shape their reputation further by enhancing their security programs, such as strict encrypted file systems and data loss prevention software, as this will also increase consumer trust in the technology. Users must be well-informed and updated about how their data is being protected. CS providers can also include those security details in the user subscription form as a reference. Users need to have a more favorable opinion of CS services, particularly in today's society, where potential users turn to social media as a resource for product and service reviews. There is a tendency for consumers to be influenced by group opinions rather than their personal beliefs.

### 4.9 Limitation and future research

Although this research met the minimum respondent requirement, the sample size can be raised further to include individuals from other regions of the Philippines. This will give a better picture of what influences Filipino consumers to use CS services. Being a benchmark on the study, a more diversified set of findings could be obtained by conducting analyses based on the demographic characteristics, socioeconomic status, and generational cohorts to which the participants belong, as well as the types of data being stored by the respondents. Also, the survey could be improved by conducting thorough screening and rephrasing any question that may generate confusion among respondents.

Qualitative research may also be employed in future studies to gain an understanding of the beliefs, attitudes, and motivations why or why not Filipinos use CS services. Filipinos can be very thrifty at times. This attitude can be incorporated into the analysis to ascertain the factors influencing the use of CS services among Filipinos.

Finally, this study can be further improved by using more advanced data analysis methods. Although SEM has consistently demonstrated its powerful analytical capabilities in numerous research articles, it is worth considering alternative approaches, such as machine learning techniques, including neural networks and random forests as they can achieve higher accuracy levels in data analysis. The number of observed variables that serve as indicators for evaluating each factor can also be expanded since machine learning algorithms can manage large quantities of data; as well as larger framework with nonlinear relationships [89].

## 5 Conclusion

As technology advances, the amount of data generated and collected also increases. This circumstance necessitates additional storage space for these data. Here, CS enters into play. CS is a service that digitally stores, remotely manages and backs up, and renders internet-accessible data. Users need not be concerned about the accessibility of their data outside of their houses or workplaces. This is because CS services can be accessed from any location and at any time, provided there is an internet connection. Even mobile phones alone can be used to access these services. It is also a sophisticated and convenient method of file exchange. For instance, rather than attaching large files to emails or passing along physical storage devices such as thumb drives, users can send their colleagues links to the files they need to access at their convenience.

However, despite the benefits of CS services, it has yet to be widely used in developing nations such as the Philippines. This study integrated two theoretical frameworks, namely the Extended Technology Acceptance Model and the Valence Framework, to better comprehend the factors that influence the behavioral intention of Filipino consumers towards adopting CS services. The factors evaluated in this study were the following: Perceived Usefulness, Voluntariness, Perceived Ease of Use, Experience, Job Relevance, Perceived Ubiquity, Perceived Benefits, Perceived Risk, Perceived Cost, Subjective Norm, Behavioral Intention, and Actual Use.

The data was obtained via an online survey, and the responses from 431 cloud users, consisting mainly of students and working individuals, were examined using Structural Equation Modeling. Based on the findings, it was found that Perceived Cost, Experience, and Voluntariness were not significant determinants of behavioral intention. Moreover, Perceived Benefit and Perceived Usefulness are observed to be the strongest determinants of Behavioral Intention to use CS services. Job Relevance was also found to be a significant factor.

CS providers could use the findings of this study as a guide on which to prioritize when improving their services. Since the perceived benefit, perceived usefulness, and job relevance influence behavioral intention, CS providers should focus more on these. They should consider additional ways to make their offerings more beneficial to students and working individuals. Furthermore, considering the substantial influence of perceived risk and subjective norms on this research, CS providers must strengthen their security measures to boost users' trust in CS technology. Consumers who receive excellent service are likely to give positive reviews, which can be helpful to individuals who are also thinking of purchasing CS for their data.

This study contributes to the little literature that employs the two aforementioned theoretical frameworks in analyzing consumer adoption of CS services in developing countries, such as the Philippines. This study can be further improved by expanding the number of observed variables that serve as indicators for evaluating each factor. More diverse results can also be obtained if participants come from a much larger geographical scale, such as regional or national. Machine learning techniques, such as neural networks and random forests, can also be employed as an alternative to structural equation modeling for higher accuracy in data analysis. Finally, although the main emphasis of this study is on CS services, this can also serve as a valuable resource when analyzing consumer intentions concerning the adoption of other novel technologies applied in various sectors, including education, e-commerce, healthcare, and more.

## Data availability

Data will be made available upon reasonable request to the corresponding author.

## CRediT authorship contribution statement

**Gerlyn C. Altes:** Writing – review & editing, Writing – original draft, Visualization, Validation, Supervision, Software, Resources, Project administration, Methodology, Investigation, Formal analysis, Data curation, Conceptualization. **Ardvin Kester S. Ong:** Writing – review & editing, Writing – original draft, Visualization, Validation, Supervision, Software, Resources, Project administration, Methodology, Investigation, Funding acquisition, Formal analysis, Conceptualization. **Josephine D. German:** Writing – review & editing, Visualization, Validation, Supervision, Software, Resources, Project administration, Methodology, Investigation, Funding acquisition.

## Declaration of competing interest

The authors declare that they have no known competing financial interests or personal relationships that could have appeared to influence the work reported in this paper.
