# Supplementary material for: Determining factors affecting Filipino consumers’ behavioral intention to use cloud storage services: An extended technology acceptance model integrating valence framework
Source: Heliyon. 2024 Feb 16;10(4):e26447. doi: 10.1016/j.heliyon.2024.e26447 (PMC10884492; doi:10.1016/j.heliyon.2024.e26447)
Supplement: Multimedia component 1 [file mmc1.docx]

**Measure Items**

| **Construct** | **Item** | **Item Detail** |
| --- | --- | --- |
| Subjective  Norm | SN1 | My boss/teacher influenced me to use cloud storage. |
|  | SN2 | My colleagues/classmates influenced me to use cloud storage. |
|  | SN3 | My family/relatives influenced me to use cloud storage. |
|  | SN4 | My friends influenced me to use cloud storage. |
|  | SN5 | My affiliation with an organization influenced my decision to utilize cloud storage. |
| Job  Relevance | JR1 | Use of cloud storage is important for my work/study. |
|  | JR2 | In terms of information / file sharing, cloud storage helps my work/study. |
|  | JR3 | In terms of data analysis, cloud storage helps my work/study. |
|  | JR4 | In terms of data reporting, cloud storage helps my work/study. |
|  | JR5 | In terms of storing information, cloud storage helps my work/study. |
| Perceived usefulness | PU1 | I think cloud storage helps me work/study effectively. |
|  | PU2 | I think using cloud storage increases my productivity. |
|  | PU3 | Cloud storage enables me to archive and retrieve my personal data faster. |
|  | PU4 | Cloud storage enhances my effectiveness in archiving and retrieving my personal data. |
|  | PU5 | I believe cloud storage simplifies my work/study. (Example: Link now replaces huge file attachment in an emails) |
|  | PU6 | Time need to upload and download data meets user’s requirement. |
| Perceived Ease  of Use | PEOU1 | Cloud storage is easy to use. |
|  | PEOU2 | It is easy to get cloud storage to do what I want it to do. |
|  | PEOU3 | Learning to operate cloud storage is easy |
|  | PEOU4 | I believe it is easy and convenient to use since I can access cloud storage services anytime even via mobile devices. |
| Experience | EX1 | I am experienced in using cloud storage. |
|  | EX2 | I have an interesting experience in using cloud storage. |
|  | EX3 | I did not have difficulties using the cloud storage. |
| Voluntariness | VO1 | I voluntarily use cloud storage. |
|  | VO2 | My boss/teacher does not require to use cloud storage. |
|  | VO3 | My work/school does not require to use cloud storage. |
|  | VO4 | My affiliated organizations do not require to use cloud storage. |
| Perceived Ubiquity | PQ1 | Use of cloud storage doesn’t interrupt my other task. (Example: Uploading/downloading files while running another program.) |
|  | PQ2 | These services allow me to access data at the best moment for me. |
|  | PQ3 | Using these services outside my home or my workplace is not a problem for me. |
|  | PQ4 | When I use these services, I can achieve things that I cannot achieve in any other way. |
| Perceived Benefits | PB1 | Recovery of data after disaster, power failure or other unfortunate scenarios is not a hindrance |
|  | PB2 | The data is saved in secured server. |
|  | PB3 | Cloud is space saving. |
|  | PB4 | I don’t worry of Server Maintenance. |
| Perceived  Risk | PR1 | There is a high potential for loss involved in using cloud provider for work / personal archiving. |
|  | PR2 | There is a considerable risk involved in using cloud storage for work / personal archiving. |
|  | PR3 | A decision to use cloud storage for work / personal archiving is risky. |
|  | PR4 | I fear of cybercrime. |
|  | PR5 | I think it is risky to give personal information when registering for cloud storage services. |
|  | PR6 | I think using cloud storage has potential risks in payment transactions. |
| Perceived  Cost | PC1 | I may encounter financial loss due to data loss. |
|  | PC2 | I may encounter financial loss due to data leaking. |
|  | PC3 | I may encounter financial loss due to unstable service. |
|  | PC4 | I am willing to pay a significant amount monthly for cloud storage consumption. |
| Behavioral  Intention | BI1 | I have the intention to use cloud storage to archive my work and personal data. |
|  | BI2 | I intend to use cloud storage for work and personal archiving in the future. |
|  | BI3 | I will use more cloud storage if I have the financial capacity. |
|  | BI4 | I plan to save up financially to continue using the cloud storage services. |
| Actual  Use | AU1 | I always use cloud storage for my daily work / studies. |
|  | AU2 | I use cloud storage more than thumb drives, external drives or other physical storage media. |
|  | AU3 | I make sure to synch my personal data and work files in cloud storage. |
